# Supplementary material for: Multi-Omics Analysis Reveals the Role of Sigma-1 Receptor in a Takotsubo-like Cardiomyopathy Model
Source: Biomedicines. 2023 Oct 12;11(10):2766. doi: 10.3390/biomedicines11102766 (PMC10604683; doi:10.3390/biomedicines11102766)
Supplement: Supplementary file 1 [file biomedicines-11-02766-s001.zip › biomedicines-2624391-supplementary/Supplementary material/Legend.pdf]

Supplementary Materials

Figure S1. PCR showing the complete deficiency of Sigmar1 in KO mice. (*n* = 2).

Figure S2. Correlation analysis between differential bacteria (top 40 at the genus level) and differential metabolites (top 40 VIP values), WT_C_VS_WT_ISO.

Figure S3. α-diversity indices between the WT_ISO and KO_ISO groups.

Table S1. The number of differential metabolites.

Table S2. KEGG pathways of differential metabolites in WT_C vs. WT_ISO.

Table S3. KEGG pathways of DEGs in WT_C vs. WT_ISO.

Table S4. KEGG pathways of differential metabolites in WT_C vs. KO_C.

Table S5. KEGG pathways of DEGs in WT_C vs. KO_C.

Table S6. KEGG pathways of differential metabolites in WT_ISO vs. KO_ISO.

Table S7. KEGG pathways of DEGs in WT_ISO vs. KO_ISO.
