# Supplementary material for: Multi-Omics Analysis Reveals the Role of Sigma-1 Receptor in a Takotsubo-like Cardiomyopathy Model
Source: Biomedicines. 2023 Oct 12;11(10):2766. doi: 10.3390/biomedicines11102766 (PMC10604683; doi:10.3390/biomedicines11102766)
Supplement: Supplementary file 1 [file biomedicines-11-02766-s001.zip › biomedicines-2624391-supplementary/Supplementary material/Supplementary material.pdf]

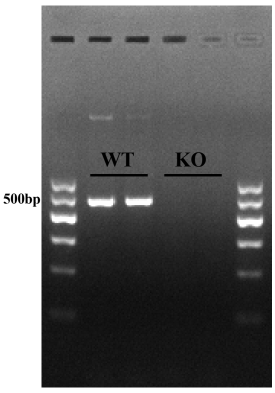

Figure S1. PCR showing the complete deficiency of Sigmar1 in KO mice. (*n* = 2)

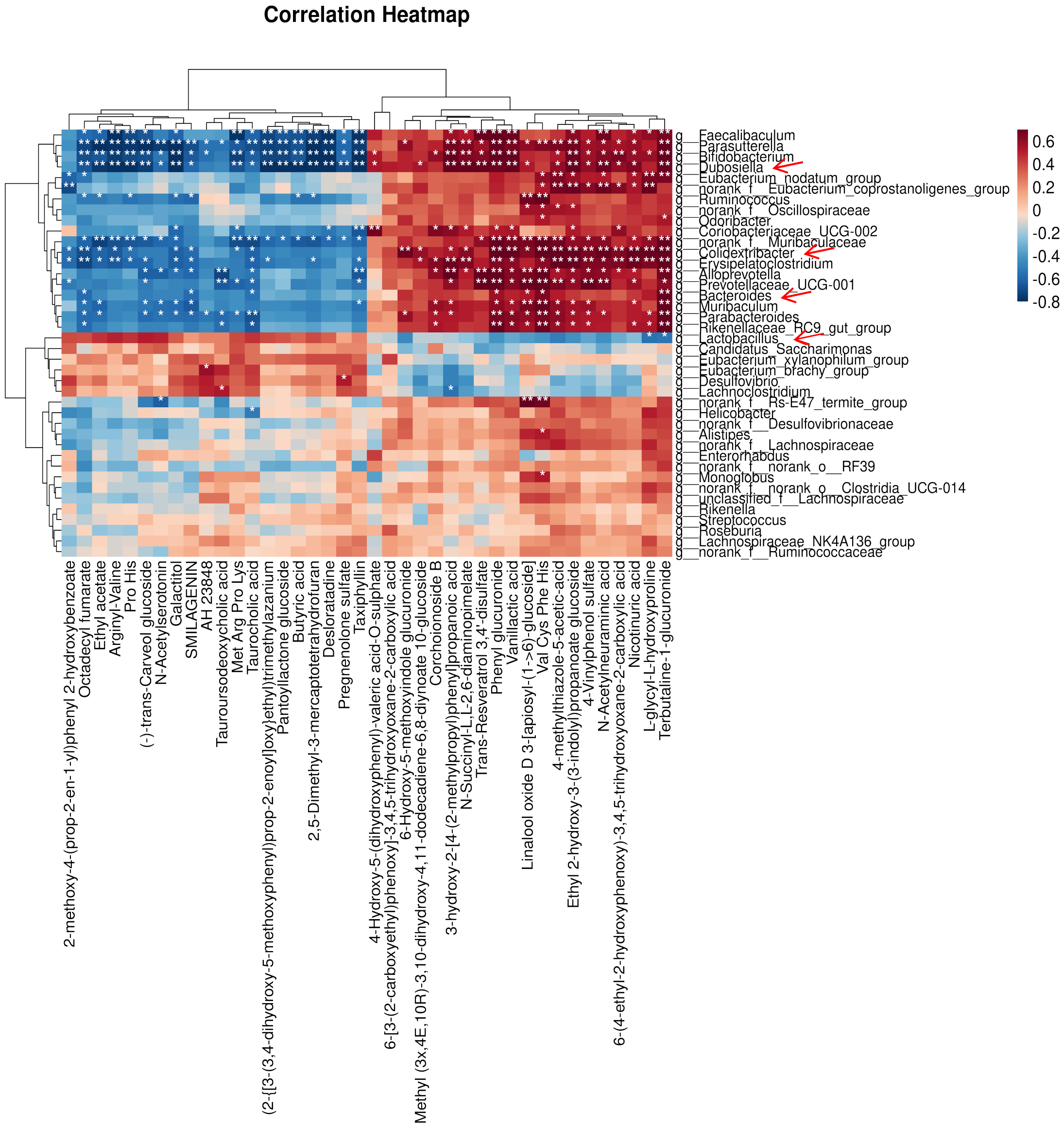

Figure S2. Correlation analysis between differential bacteria (top 40 at the genus level) and differential metabolites (top 40 VIP values), WT_C_VS_WT_ISO.

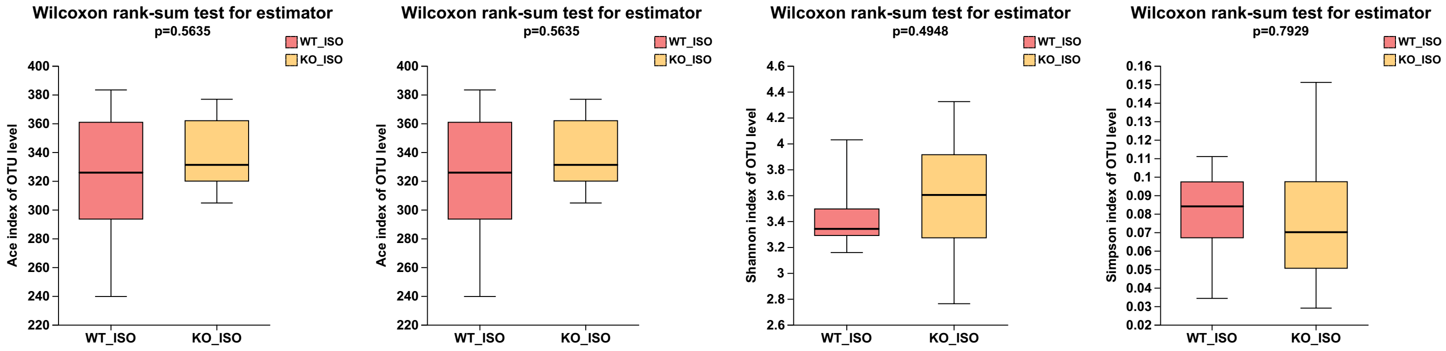

Figure S3. α-diversity indices between the WT_ISO and KO_ISO groups.

Differential metabolites statistics

	up	down
WT_C_VS_WT_ISO	135	58
WT_C_VS_KO_C	113	50
WT_ISO_VS_KO_ISO	123	176

Table S1. The number of differential metabolites.
